# Seminal Fluid Metabolomic Markers of Oligozoospermic Infertility in Humans

**DOI:** 10.3390/metabo10020064

**Published:** 2020-02-11

**Authors:** Federica Murgia, Valentina Corda, Marianna Serrenti, Valeria Usai, Maria Laura Santoru, K. Joseph Hurt, Mauro Passaretti, Maria Carla Monni, Luigi Atzori, Giovanni Monni

**Affiliations:** 1 Clinical Metabolomics Unit, Department of Biomedical Sciences, University of Cagliari, 09121 Cagliari, Italy; federica.murgia@unica.it (F.M.); marialaurasantoru@gmail.com (M.L.S.); mauro.passaretti@gmail.com (M.P.); latzori@unica.it (L.A.); 2 Department of Prenatal and Preimplantation Genetic Diagnosis and Fetal Therapy, Ospedale Pediatrico Microcitemico “A.Cao”, 09121 Cagliari, Italy; cordavale@hotmail.it (V.C.); mariannaserrenti@tiscali.it (M.S.); valeriau@tiscali.it (V.U.); mariacarlamonni@live.it (M.C.M.); 3 Divisions of Maternal Fetal Medicine and Reproductive Sciences, Department of Obstetrics and Gynecology, University of Colorado Anschutz Medical Campus, Aurora, CO 80045, USA; k.joseph.hurt@cuanschutz.edu

**Keywords:** seminal plasma, oligozoospermia, metabolomics, nuclear magnetic resonance, biomarkers

## Abstract

Infertility affects 12–15% of couples worldwide, and male factors are the cause of nearly half of all cases. Studying seminal fluid composition could lead to additional diagnostic accuracy and a better understanding of the pathophysiology of male factor infertility. Metabolomics offers a new opportunity to evaluate biomarkers and better understand pathological mechanisms. The aim of the study was to identify new markers or therapeutic targets to improve outcomes in male factor or idiopathic infertility patients. Semen samples were obtained from 29 men with a normal spermogram test, and from 18 oligozoospermic men. Samples were processed and analyzed by Nuclear Magnetic Resonance spectroscopy and, subsequently, multivariate and univariate statistical analyses. Receiving Operator Curves (ROC) and Spearman correlations were also performed. An Orthogonal Partial Least Square Discriminant Analysis supervised multivariate model was devised to compare the groups. The levels of fructose, myo-inositol, aspartate and choline were altered. Moreover, Spearman Correlation associated fructose, aspartate and myo-inositol with the total amount of spermatozoa, total motile spermatozoa, % of immotility and % of “in situ” spermatozoic motility respectively. NMR-based metabolomics allowed the identification of a specific metabolic fingerprint of the seminal fluids of patients affected by oligozoospermia.

## 1. Introduction

Infertility affects 12–15% of couples worldwide, with profound psychological, social and economic consequences. A male factor is the cause of nearly half of all infertility cases [1]. Non-pregnancy due to male causes is diagnosed by seminal fluid analysis, which allows us to obtain information about the functional state of the seminiferous tubules, epididymis and accessory sex glands. The World Health Organization (WHO) has determined the criteria for normal seminal fluid characteristics [2] and classifies the forms of male infertility by evaluating various parameters such as sperm count, motility and morphology. Considering the cut-off values of these parameters, the WHO identifies descriptive frameworks of various pathological states: oligozoospermia, astenozoospermia, theratozoospermia and combinations of the three [3,4]. Numerous studies show a correlation between reduced sperm count and non-pregnancy, showing evidence of the predictive value of sperm count [5,6]. There is some debate as to whether semen analysis alone can adequately assess male fertility. The standardization of procedures is essential, due to differences in results bringing the usefulness and accuracy of the test into question [7,8]. Furthermore, in 10–20% of infertility cases no cause is identified, and a portion of those cases is likely due to male factors as well [9,10].

Seminal fluid contains a variety of molecules produced primarily by the accessory sex glands, but also by spermatozoa, and their precursors. This complex media supports not only sperm survival and health but also optimal conditions for fertilization within the female reproductive tract [11]. Thus, the study of seminal fluid composition for markers associated with infertility could lead to additional diagnostic accuracy and a better understanding of the pathophysiology of male factor infertility. The necessity to improve current knowledge on the molecular and cellular basis of the functionality of sperm is evident. Improving the ability to diagnose, and facilitating the study of abnormal and/or dysfunctional cells, is important for developing a possible therapy. Currently, despite numerous and in-depth studies of male infertility, there is currently no effective therapy, bar the use of medically assisted reproduction, debated by several authors [10].

One approach to identifying novel biomarkers of disease uses metabolomics in a combination of analytical and biochemical techniques [12]. Nuclear magnetic resonance spectroscopy (NMR) and mass spectrometry (MS) are the most frequently employed techniques in metabolomics and are powerful methods for differentiating the diversities between cases and controls [13,14]. Bioinformatics increases the value of metabolomic analysis by assessing general biochemical and signaling pathways that may be altered in disease states [15]. To date, few metabolomics studies for human male factor infertility have been reported [16,17,18,19]. Therefore, we performed an NMR metabolomic analysis of human seminal plasma from normal fertile controls and men with oligospermic infertility. Our goal was to identify new markers or therapeutic targets to improve outcomes in male factor or idiopathic infertility patients. We hypothesized that specific metabolic pathways may be abnormal in oligospermic seminal fluid.

## 2. Methods

### 2.1. Sample Selection

This prospective study was conducted in the Department of Prenatal and Preimplantation Genetic Diagnosis and Foetal Therapy, Ospedale Pediatrico Microcitemico A.Cao, (Cagliari, Italy). Written consent was obtained from all participating men and the study was approved by the Institutional Review Board of Microcitemico Hospital. Moreover, the study was conducted in accordance with the Declaration of Helsinki. Human ejaculates were obtained through masturbation after 3–4 days of sexual abstinence. A portion of the sample was used for routine semen analysis (ejaculate volume, sperm concentration, morphology assessment, motility and total progressive motile sperm count). The morphology was assessed using the Papanicolau stain. The motility assessment was made using the Makler chamber, first counting the motile elements by row and column and then the immotile elements. The respective motility percentages and the total count were then carried out. To perform a more accurate evaluation, the motility of the spermatozoa was subsequently evaluated, placing 20 µL of the sample on a slide and evaluating 200 spermatozoa at 200× [20,21]. All parameters refer to the World Health Organization (WHO) manual 2010, V edition [22]. Semen samples were obtained from 29 men with a proven normal spermogram test (control group, C) as assessed by WHO Manual for Semen Analysis (2010): (Total spermatozoa (10^6^ )  ≥  39, sperm concentration (10^6^  mL^−1^)  ≥  15, sperm morphology (normal forms, %)  ≥ 4, total motility (%) ≥ 40) and from 18 oligozoospermic men. All the subjects were referred to our centre for infertility treatment. The main criterion for classification of oligozoospermic men was low sperm quantity (<15 million spermatozoa/mL, <40% motile spermatozoa, <4% normal forms). Ejaculate specimens were deposited directly in sterile microurine jars. The clinical parameters of the enrolled patients are reported in Table 1.

### 2.2. Sample Preparation

500 µL of all the samples were centrifuged for 10 min at 12.052× *g* at 4 °C. The supernatant was collected for metabolomic analysis. 400 µL of seminal fluid was mixed with 600 µL of methanol, 400 µL of Milli-Q water and 600 µL of chloroform [23], then vortexed for 1 min and centrifuged at 4500 rpm, 15 min 4 °C. 750 µL of the supernatant was used for the NMR analysis. Aliquots (10 µL) from each sample were used to create a pool for quality control (QC) samples. The QC sample was analyzed at the beginning and at the end of the analysis.

### 2.3. Nuclear Magnetic Resonance Analysis and Data Processing

For the NMR analysis, 600 µL of the water-phase, containing low-weight molecules (amino acids, sugars, etc.) for each sample, were dried overnight in a speed-vacuum. The dried water-phase was re-suspended in 697 µL of phosphate buffer 100 mM in D_2_O, pH 7.3 and 3 µL of trimethylsilyl-propanoic acid (TSP) 5.07 mM.TSP was added to provide an internal reference for the chemical shifts (0 ppm), and 650 µLof the solution were transferred to a 5 mm NMR tube.

The samples were analyzed with a Varian UNITY INOVA 500 spectrometer (Agilent Technologies, Inc., Santa Clara, CA, USA), which was operated at 499 MHz, equipped with a 5 mm triple resonance probe with *z*-axis pulsed field gradients, and with an auto-sampler with 50 locations. One-dimensional ^1^H-NMR spectra were collected at 300 K with a pre-sat pulse sequence. The spectra were recorded with a spectral width of 6000 Hz, a frequency of 2 Hz; an acquisition time of 1.5 s, a relaxation delay of 2 ms, and a 90° pulse of 9.5 µs. The number of scans was 256. Each Free Induction Decay (FID) was zero-filled to 64 k points and multiplied by a 0.5 Hz exponential line broadening function. The spectra were manually phased and baseline corrected. By using MestReNova software (version 8.1, Mestrelab Research S.L. Santiago de Compostela, Spain) each NMR spectrum was divided into consecutive “bins” of 0.04 ppm. The spectral area investigated was the region between 0.8 and 8.6 ppm. To minimize the effects of the different concentrations of seminal plasma samples, the integrated area within each bin was normalized to a constant sum of 100. The final data set consisted of a 161 × 47 matrix.

### 2.4. Statistical Analysis

Multivariate statistical analysis was performed on NMR data by using SIMCA-P software (ver. 15.0, Sartorius Stedim Biotech, Umea, Sweden) [24]. The variables were Pareto scaled.

The initial data analyses were conducted using the Principal Component Analysis (PCA) for the exploration of the sample distributions without classification. In particular, PCA analysis was performed to observe intrinsic clusters and find outliers. To identify potential strong outliers, the Hotelling’s T2 test was applied. Moreover, the PCA model was performed including the QC samples and, based on their tight clustering, it showed a good quality of the analysis in our batch. Subsequently, a supervised analysis was applied. Orthogonal Partial Least Square (OPLS-DA) analysis was employed to discriminate between patients with oligozoospermia and healthy patients, maximizing the discrimination between samples assigned to different classes. The OPLS-DA model removes variability not relevant to class separation [25,26].

The variance and the predictive ability (R^2^X, R^2^Y, Q^2^) were established to evaluate the suitability of the models. In addition, to avoid OPLS-DA-derived model over-fitting, permutation tests with 400 iterations using the 7-fold cross-validation method were performed. In short, this rigorous test compares the goodness of fit of the original model with that of randomly permuted models [27]. The permutation test provides model validity in terms of the explained variance parameter (R^2^) and the cross-validation parameter (Q^2^) that indicates the goodness of fit and accuracy of prediction, respectively, in the supervised model. In parallel, CV-ANOVA (analysis of variance testing of cross-validated predictive residuals) tests were performed to determine significant differences between oligozoospermia and the healthy control group in the OPLS-DA models (*p* < 0.05). To evaluate the predictive and diagnostic ability of the model, external validation was carried out. About half of the total samples (15 healthy controls and nine oligozoospermias) were randomly selected from each group as the test set, and the remaining part of the samples (14 healthy controls and nine oligozoospermias) constituted the training set for validation. An ROC curve was created using the cross-validated predicted Y-values of the ^1^H-NMR OPLS-DA data.

Variables corresponding to a VIP (Variables Important in the Projection) value of >1 (a measure of their relative influence on the model, see supplementary material) from the OPLS-DA model together with the Volcano plot (plot constructed with the VIP value versus p(corr)), were indicated as the most significant in discriminating between the metabolic profiles of oligozoospermia and healthy controls. Indeed, VIPs of >1 are the most relevant for explaining Y (assignment of two classes). To study a possible linear relationship between the metabolic profile (matrix X, predictor variables, e.g., metabolites) and the clinical parameters (matrix Y, dependent variable, age, motility of spermatozoa, progressive motility and % of “in situ” motility), PLS projection to latent structures regression models were carried out [28].

Subsequently, the variables were identified using the Chenomx NMR Suite 7.1 (Chenomx Inc., Edmonton, Alberta, Canada) [29] and literature data. GraphPad Prism software (version 7.01, GraphPad Software, Inc., San Diego, CA, USA) was used to perform the univariate statistical analysis of the data resulting from the multivariate analysis. To verify the significance of the resulting metabolites, a U-Mann Whitney test was performed, followed by ROC curves to test the sensitivity and specificity of the metabolites with *p*-values <0.05. ROC curves are conventionally used to evaluate diagnostic performance in clinical research. Finally, once the significant metabolites were selected, a Spearman Correlation was performed between each selected significant metabolite and clinical parameters (total spermatozoa, total motile spermatozoa, age, % of motility of spermatozoa, % of progressive motility and % of “in situ” motility).

## 3. Results

The sample included 47 subjects, with 29 men having a normal seminal analysis (C, control), and 18 men demonstrating oligozoospermia (P, pathological).

**Figure 1:**
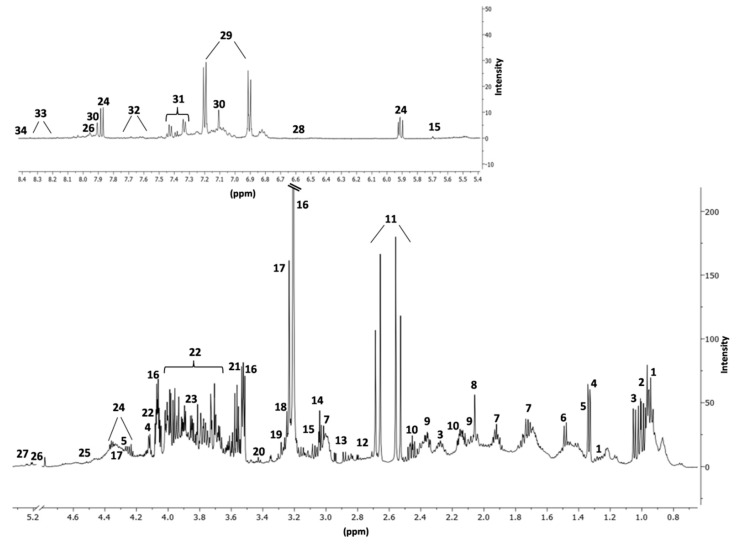
Identified compounds in a control sample seminal fluid NMR spectrum. * = uncertain attribution; 1. Isoleucine; 2. Leucine; 3.Valine; 4. Lactate; 5. Threonine; 6. Alanine; 7. Lysine; 8. N-Acetyl groups; 9. Glutamate; 10. Glutamine; 11. Citrate; 12. Aspartate; 13. Asparagine; 14. Creatine; 15. Cis-Aconitate; 16. Choline; 17. sn-Glycero-3-Phosphocholine; 18. Betaine; 19. Myo-Inositol; 20. Taurine; 21. Glycine; 22. Fructose; 23. Serine; 24. Uridine; 25. Ascorbate; 26. N-Acetyl-Glucosamine*; 27. Glucose-6-Phosphate; 28. Trans-Aconitate; 29. Tyrosine; 30. Histidine; 31. Phenylalanine; 32. Tryptophan; 33. Adenosine; 34. Formate.

Semen samples were analyzed with NMR, and the number of metabolites identified was 34 (Figure 1). A representative 500 MHz one-dimensional ^1^H NMR spectra, resulting from seminal plasma samples from a healthy control and a patient with oligozoospermia, is reported in Figure S1.

**Figure 2:**
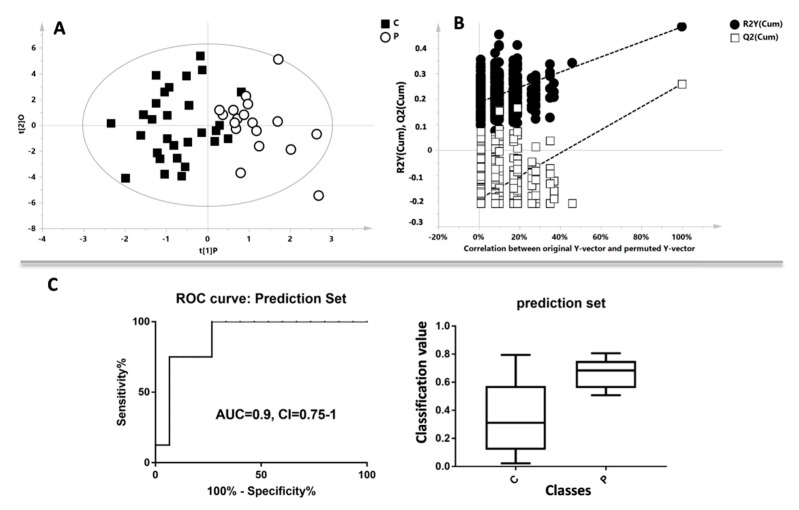
Multivariate analysis between controls patients (C) and oligozoospermic patients (P). (**A**) Supervised Orthogonal Partial Least Square (OPLS-DA) model, black boxes represent the C class, while white circles represent the P class. (**B**) Permutation test of the OPLS-DA model. (**C**) Results of the prediction set analysis: ROC curve based on the cross-validated predicted Y-values, and the respective box plot.

The non-supervised multivariate PCA was applied; the obtained score plot and result of the T^2^ Hotelling test indicated the presence of one outlier. The PCA model with the QC samples is reported in Figure S2. A separation of the samples, in line with the presence of oligozoospermia, was subsequently observed by the application of the supervised OPLS-DA model (R^2^X = 0.723; R^2^Y = 0.632; Q^2^ = 0.417, *p* = 0.004, Figure 2A) with the respective permutation test (Figure 2B). The cumulative values of total Y-explained variance (R^2^) and the Y-predictable variation (Q^2^) values indicated proper modelling. Additionally, to test the power of the model, about half of the total samples (15 healthy controls and nine oligozoospermias) were randomly selected from each group and the corresponding cross-validated predicted Y-values were used to create the ROC curve (Figure 2C, AUC = 0.9, CI = 0.75–1, *p* = 0.002). The respective box plot indicates the difference of the values based on the classes of samples (*p* = 0.0009).

**Figure 3:**
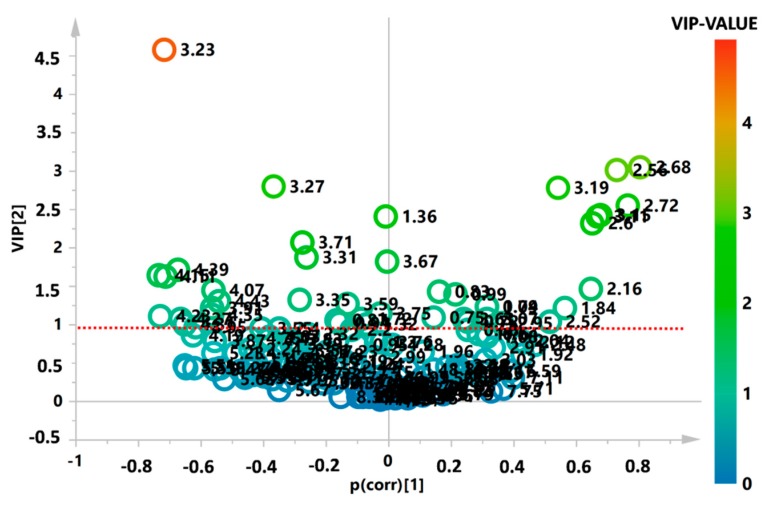
V-plot of the supervised OPLS-DA model, indicating the importance of the variables based on their position in the plot and their VIP-value. Variables with a value of > 1 were considered the most relevant.

Subsequently, the most important variables were identified through the analysis of the V-plot and using the corresponding VIP-value. Variables with a value of >1 were considered the most relevant (Figure 3).

**Figure 4:**
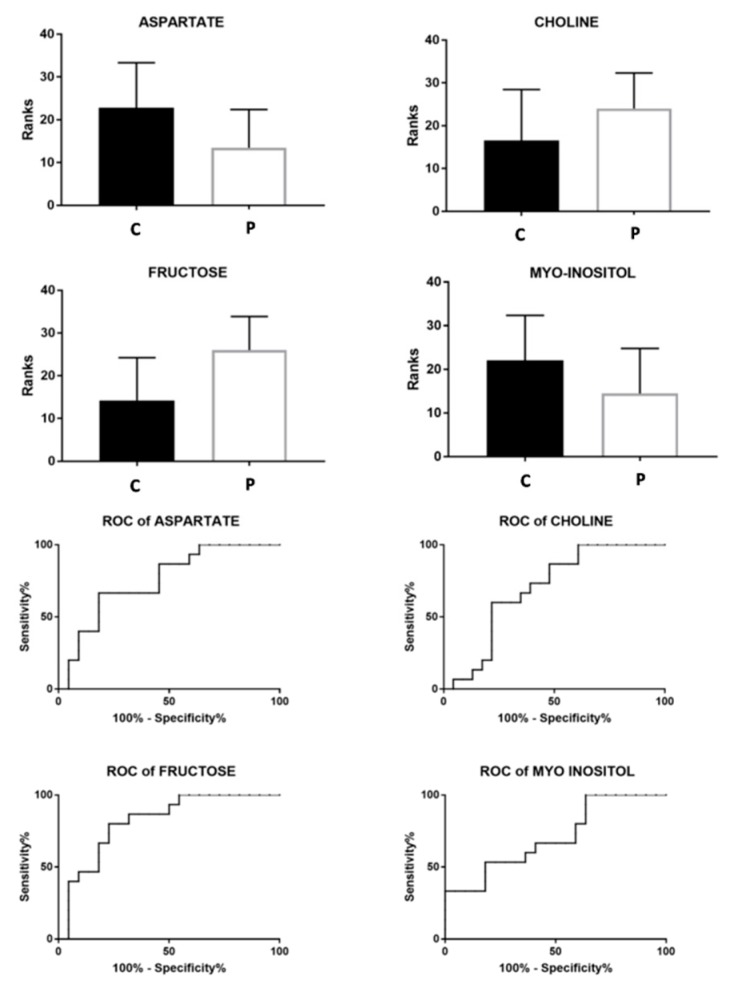
Bar graphs and ROC curves of the metabolites exhibiting a *p*-value of < 0.05. U-Mann Whitney analysis was used. C represents the control class while P represent the oligozoospermic patients.

These variables, with a VIP-value of >1, were identified and underwent the univariate statistical analysis with the U-Mann Whitney test. Fructose, choline, aspartate and myo-inositol were the metabolites that exhibited the greatest differences between the studied groups according to a *p*-value of <0.05 and were selected to create the ROC curve. The bar-graphs and the corresponding ROC curve of these metabolites are reported in Figure 4 and the corresponding statistical parameters are reported in Table 2.

To test a possible linear correlation between the whole metabolic profile of the patients and clinical parameters such as age, % of motility of spermatozoa, % progressive motility and % of “in situ” motility, PLS models were performed. The analysis showed no linear correlation with all the parameters, indeed, the R^2^ values were <0.2 for each model (Figure S3).

**Figure 5:**
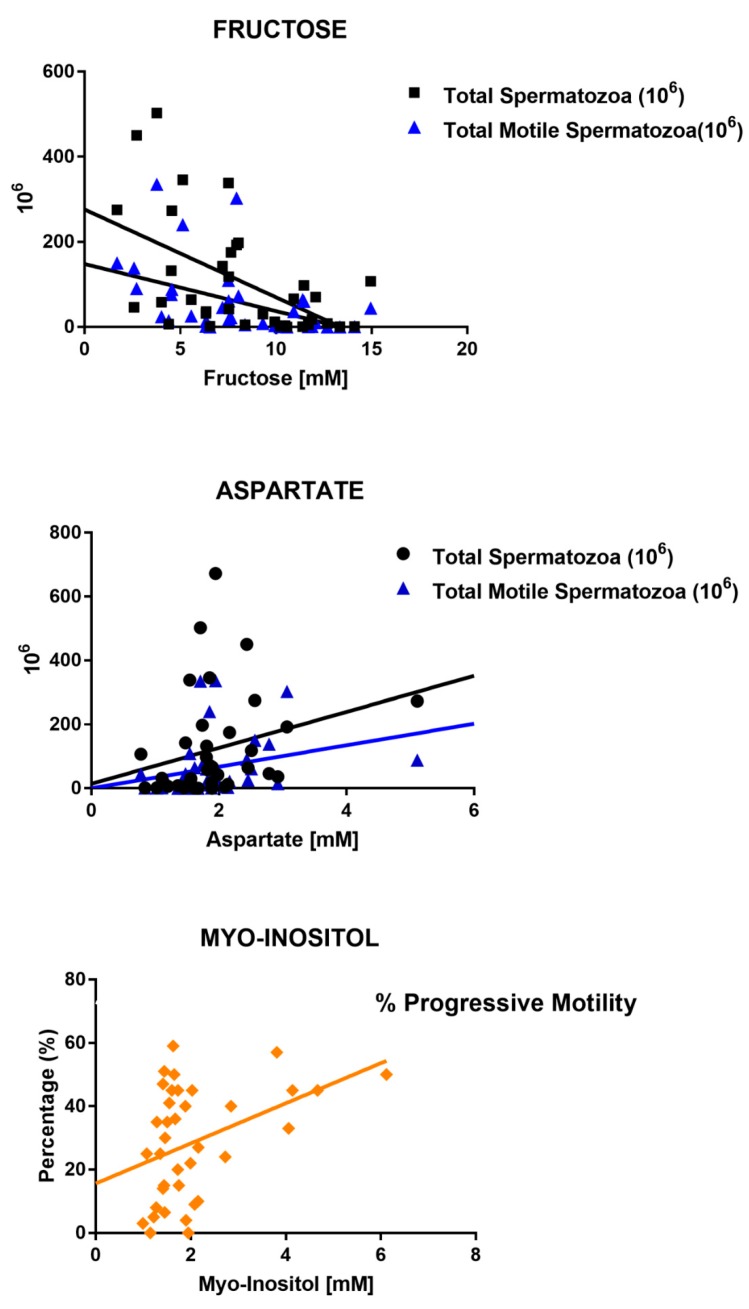
Plots showing a correlation between the different metabolites tested, and the clinical parameters of the patients: total spermatozoa, total motile spermatozoa, % of motility of spermatozoa, % of progressive motility having *p*-value <0.05. Spearman correlation was used.

Finally, the Spearman correlation was applied to fructose, choline, aspartate and myo-inositol and the following clinical parameters: total spermatozoa, total motile spermatozoa, age, % of motility of spermatozoa, % of progressive motility and % of “in situ” motility”. In Figure 5, correlation plots for the metabolites and clinical parameters having a *p*-value of <0.05 are shown, while Table 3 indicates which correlation tests reached statistical significance.

## 4. Discussion

The identification and quantification of biomarkers were achieved through a combination of analytical, biochemical and spectral analysis, thereby establishing signatures of the metabolites for healthy control populations and cases with specific illnesses. The simplest and fastest approaches to distinguish oligozoospermic men from controls utilize classification methods, resulting in a predictive model that can categorize each man based on his NMR spectra, creating unique metabolome profiles that help to differentiate patients from controls. Moreover, the metabolomics approach allowed the identification of a set of specific metabolites which have been tested as biomarkers with the analysis of sensitivity and specificity. In our data, aspartate, choline, fructose and myo-inositol (MYO) were identified as the most relevant to classify and characterize men affected by oligozoospermia. Finally, the metabolites were correlated with clinical parameters of the patients, such as total spermatozoa, total motile spermatozoa, age, % of motility of spermatozoa, % of progressive motility, and % of “in situ” motility to investigate a possible association. To the best of our knowledge, only a few studies have been performed with the metabolomics approach [30] on a cohort of patients affected by oligozoospermia and, for this reason, it is not simple to explain the biological meaning of the metabolites by comparing our study with others. Zhao et al. applied an untargeted GC-MS analysis on patients affected by idiopathic asthenozoospermia, and they found a different panel of altered metabolites compared with our study based on the different analytical technique used [31]. Moreover, Jayaraman et al. performed an NMR analysis of seminal fluids of patients with different forms of male infertility, highlighting the presence of a difference in biochemical profiles, with a specific emphasis on idiopathic infertility where the level of lysine could be of diagnostic use [17].

In our analysis, fructose played a leading role in the separation of the two classes. It is known that the seminal vesicles produce and secrete reducing substances (fructose, ascorbic acid, ergothioneine) [32]. Fructose is essential for spermatozoa metabolism and motility as an energy source. For this reason, fructose is routinely assayed in almost all laboratories of andrology and could be used as a marker of the function of seminal vesicles. In our data, higher concentrations of fructose were found in oligozoospermic patients compared to control patients. Gonzales et al. found similar results comparing the same classes of patients [33]. In all likelihood, due to the low number of spermatozoa in the seminal fluids of oligozoospermic patients, fructose was found to be increased because it was not employed as fuel for motility of spermatozoa. It is demonstrated also by the results of the Spearman correlation where fructose appeared negatively correlated with the total number of spermatozoa and the total motile spermatozoa. Discordant results were found in literature: in particular, seminal fructose content correlated inversely with sperm count but not with sperm motility [34].

Myo-inositol is a biomolecule particularly studied in the field of medically assisted reproduction as it regulates a wide range of cellular processes (gamete development, oocyte maturation, fertilization and early embryonic development) [35]. Indeed, an increasing number of studies have supported the physiological and therapeutic role of MYO in human reproductive functions [36,37]. Previous evidence has suggested a possible role of MYO in spermatogenesis and sperm function. In animal models, a lower concentration of MYO within the epididymis has been associated with reduced fertility [38] and moreover, in humans, the enzyme MYO monophosphatase-1 is present in greater amounts in spermatozoa from asthenozoospermic samples [39]. Interesting, Colone et al. [40] recently showed that the spermatozoa of patients with oligo-astheno-teratozoospermia (OAT) are covered by amorphous fibrous material that increases seminal fluid viscosity and reduces sperm motility. Moreover, the mitochondria in the intermediate tract of spermatozoa of patients with OAT showed damaged cristae. Surprisingly, after incubation with inositol, the amorphous fibrous material disappeared and cristae damage decreased. Thereafter, spermatozoa from patients with OAT appeared similar to those obtained from normozoospermic men [40]. In line with our results, Condorelli at al. [41] found that MYO significantly improved the total and progressive motility of the sperm from the patients with OAT, while these parameters were unaffected in the spermatozoa of normozoospermic men. Incubation with MYO improved sperm progressive motility and doubled the concentration of motile spermatozoa [41]. Furthermore, MYO concentration increases with movement of the spermatozoa through epididymis and deferent duct [42].

Moreover, relating to the role of MYO in male reproduction, Chauvin et al. demonstrated that MYO concentration in the seminiferous tubules is higher than in serum [43], highlighting its fundamental role in this anatomical area, especially during reproduction [44].

The other significant metabolite change in oligozoospermic patients was choline. In line with our result, Zhang et al. [45] found in the seminal plasma of patients with asthenozoospermia relatively higher levels of phospholipids intermediates including choline, phosphocholine and glycerophosphocholine. Choline and its derivatives form important cell membrane constituents and play a pivotal role in lipid cholesterol transport and metabolism. In our study, the altered levels of choline in the seminal plasma of patients with oligozoospermia compared to control class suggested an altered choline metabolism associated with this pathological condition. Muncu et al. [46] analyzed a cohort of patients with oligoasthenoteratozoospermia (OAT) by high-resolution ^1^H NMR spectroscopy to reveal the metabolomic changes of seminal plasma compared to a control class. The levels of citrate, choline, spermine, putrescine, α-ketoglutaric acid, valine, and tyrosine were significantly different between the two groups. Further to this, levels of lactate, creatinine, lysine, arginine, and glutamine were also statistically significant. In particular, they found a decreased concentration of choline in OAT patients, in contrast with our findings.

Aspartate was found to decrease in patients affected by oligozoospermia. Moreover, in our analysis, it correlated positively with the total amount of spermatozoa and the total motile spermatozoa. High concentrations of aspartate have been found in Leydig cells [47,48] in rat testis fluid and in epididymal spermatozoa [49]. Interestingly, it was found that, in humans, a considerable amount of aspartate is present in seminal plasma and in spermatozoa, and its concentration in seminal plasma from oligoasthenoteratospermic donors is significantly reduced when compared to normospermic donors. Moreover, the concentration of aspartate in the spermatozoa reflects that found in seminal plasma [50]. The hypothesis proposed by this cited work could also be linked to our results; aspartate occurs in seminal plasma and spermatozoa in relation to the quality of the semen. Because in normospermic patients the concentration of aspartate is significantly higher than that of oligozoospermic patients, the hypothesis is that aspartate could have a specific role in spermatogenesis. In fact, it is possible that the amino acid could be implicated in the maturation and fertility of the spermatic cells.

In conclusion, the NMR-based metabolomics approach allowed the identification of a specific metabolic fingerprint of the seminal fluids of patients affected by oligozoospermia. The identification of a panel of metabolites (fructose, choline, aspartate and myo-inositol) proved useful in describing this pathological state and may provide new avenues for the management of this serious condition. Considering the absence of studies focussing on metabolic changes in the field of male sterility, further investigation is necessary.

## Figures and Tables

**Table 1:** Clinical parameters of the patients enrolled in the study.

Cohort		
	Control	Oligozoospermia
N	29	18
AGE (mean ±SD)	38.9 ± 7	38.8 ± 6
Total Spermatozoa (10^6^) (mean)	172.5	48.6
MotileSpermatozoa (10^6^) (mean)	92.4	24.5
Motility % (mean ± SD)	44.5 ± 1.5	26.8 ± 23
Progressive Motility % (mean ± SD)	18.6 ± 9	6.7 ± 10
% of “in situ” motility (mean ± SD)	7.7 ± 3	10 ± 8

**Table 2:** Statistical parameters of the Receiving Operator Curves (ROC) curves of the metabolites exhibiting *p*-value < 0.05.

	Seminal Fluid							
	Metabolites	C	P	*p*-Value	ROC-Curve			
					AUC	Std. Error	CI	*p*-Value
NMR	ASPARTATE	+	−	0.009	0.76	0.08	0.6–0.9	0.01
	CHOLINE	−	+	0.04	0.7	0.08	0.53–0.86	0.04
	FRUCTOSE	−	+	0.0008	0.82	0.07	0.67–0.94	0.001
	MYO-INOSITOL	+	−	0.03	0.71	0.08	0.54–0.88	0.03

**Table 3:** Summary of the statistical parameters of the Spearman correlation of fructose, choline, aspartate and myo-inositol with the clinical parameters of the patients. Bold values indicated significant parameters.

	Parameters	Age	Total Spermatozoa (10^6^)	Total Motile Spermatozoa (10^6^)	% Motility	% Progressive Motility	% of “In Situ” Motility
**FRUCTOSE**	**r**	−0.21	**−0.53**	**−0.59**	−0.23	−0.32	0.02
	**CI**	−0.51/0.13	**−0.73/−0.23**	**−0.77/−0.31**	−0.53/0.11	−0.6/0.01	−0.31/0.36
	***p*-Value**	ns	**0.0009**	**0.0001**	ns	ns	ns
**CHOLINE**	**r**	0.07	−0.22	−0.32	−0.03	−0.07	0.09
	**CI**	−0.26/0.4	−0.52/0.11	−0.59/0.01	−0.36/0.3	−0.39/0.27	−0.25/0.40
	***p*-Value**	ns	ns	ns	ns	ns	ns
**ASPARTATE**	**r**	−0.09	**0.40**	**0.44**	0.14	0.30	−0.05
	**CI**	−0.40/0.25	**0.08/0.64**	**0.12/0.67**	−0.19/0.45	−0.04/0.57	−0.38/0.28
	***p*-Value**	ns	**0.01**	**0.006**	ns	ns	ns
**MYO-INOSITOL**	**r**	0.24	0.33	0.21	0.29	**0.34**	−0.06
	**CI**	−0.09/0.53	−0.00/0.60	−0.12/0.50	−0.05/0.56	**−0.01/0.59**	−0.38/0.27
	***p*-Value**	ns	ns-	ns	ns	**0.04**	ns

## Supplementary Materials

The following are available online at https://www.mdpi.com/2218-1989/10/2/64/s1, Figure S1: Comparison of a representative 500 MHz one-dimensional 1H NMR spectra recorded for seminal plasma samples from a healthy control and a oligozoospermic patient, Figure S2: PCA model of NMR samples of seminal fuid of control patients and patients affected by oligozoospermia Figure S3: PLS correlation analysis between the metabolic profile of the patients and clinical parameters.
