# Supplementary material for: Seminal Fluid Metabolomic Markers of Oligozoospermic Infertility in Humans
Source: Metabolites. 2020 Feb 11;10(2):64. doi: 10.3390/metabo10020064 (PMC7074256; doi:10.3390/metabo10020064)
Supplement: Supplementary file 1 [file metabolites-10-00064-s001.pdf]

Supplementary Materials

Seminal Fluid Metabolomic Markers of Oligozoospermic Infertility in Humans

Federica Murgia[1], Valentina Corda [2], Marianna Serrenti[2], Valeria Usai[2], Maria Laura Santoru[1], K. Joseph Hurt[3], Mauro Passaretti[1], Maria Carla Monni[2], Luigi Atzori[1] and Giovanni Monni[2,*]

[1] Clinical Metabolomics Unit, Department of Biomedical Sciences, University of Cagliari, 09121 Cagliari, Italy; federica.murgia@unica.it (F.M.); marialaurasantoru@gmail.com (M.L.S.); mauro.passaretti@gmail.com (M.P.); latzori@unica.it (L.A.)
[2] Department of Prenatal and Preimplantation Genetic Diagnosis and Fetal Therapy, Ospedale Pediatrico Microcitemico "A.Cao", 09121 Cagliari, Italy; cordavale@hotmail.it (V.C.); mariannaserrenti@tiscali.it (M.S.); valeriau@tiscali.it (V.U.); mariacarlamonni@live.it (M.C.M.)
[3] Divisions of Maternal Fetal Medicine and Reproductive Sciences, Department of Obstetrics and Gynecology, University of Colorado Anschutz Medical Campus, Aurora, CO 80045, USA; k.joseph.hurt@cuanschutz.edu
* Correspondence: prenatalmonni@tiscali.it

Material and Method

VIP (Variables Important in the Projection) value referred to the variables and we considered only that having value >1 (a measure of their relative influence on the model). In the projection techniques, a strategy for selecting descriptors is provided by analyzing the weights used to construct the scores. In fact, if the weight of a variable is higher, the variable will have an important effect in the model. Viceversa, if weight approaches zero, the variable will be irrelevant and can be excluded. A useful parameter for the selection are the VIPs. Each variable of block x is associated with a parameter (VIP) which establishes the influence of that variable on the model. The VIP parameter is calculated by combining the weight that a given variable has in each projection and the explanatory power of that component of the model. The higher VIP indicates the greater influence of the variable on the model. Once a limit has been set (usually 1.0 or 0.80), all descriptors with a lower VIP are eliminated from the constructed model and a new model is calculated. Generally, this selection method improves the qualities of the models in terms of Q^2 making the model more robust in prediction.

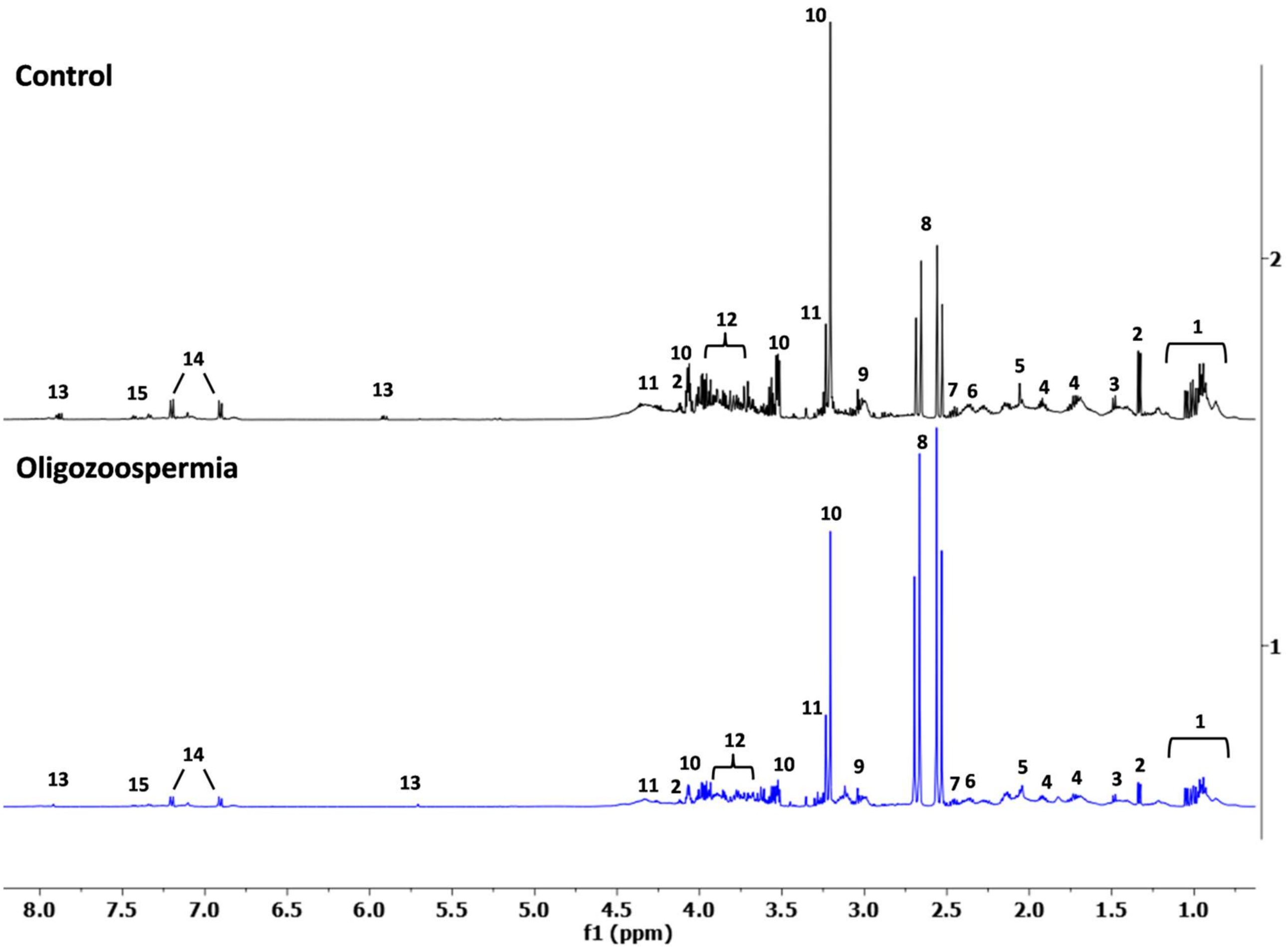

Figure S1. Representative 500 MHz one-dimensional ^{1}H NMR spectra recorded for seminal plasma samples from a healthy control (black) and a patient with oligozoospermia (blue). The numbers in the figure correspond to the major peaks: 1. Branched Chain Aminoacids; 2. Lactate; 3. Alanine; 4. Lysine; 5.N-Acetyl groups; 6. Glutamate; 7. Glutamine; 8. Citrate; 9. Creatine; 10. Choline; 11. sn-Glycero-3-Phosphocholine; 12. Fructose; 13. Uridine; 14. Tyrosine; 30. Histidine; 15. Phenylalanine.

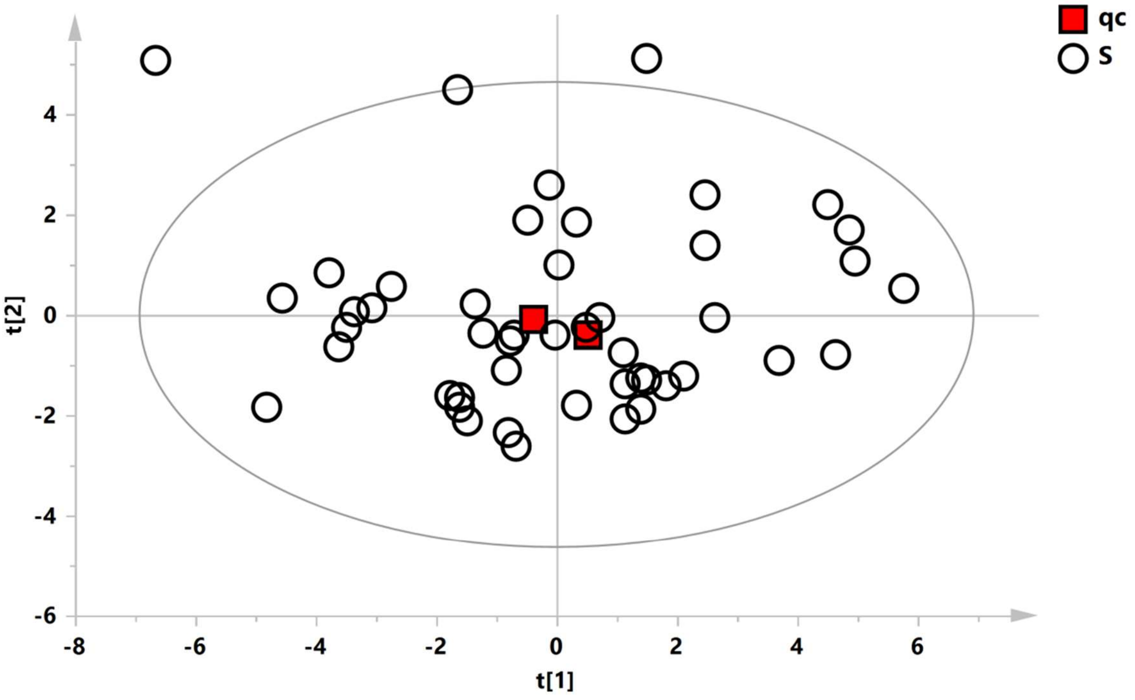

Figure S2. PCA model of NMR samples of seminal fuid of control patients and patients affected by oligozoospermia (white circles). Red boxes indicate the QC samples.

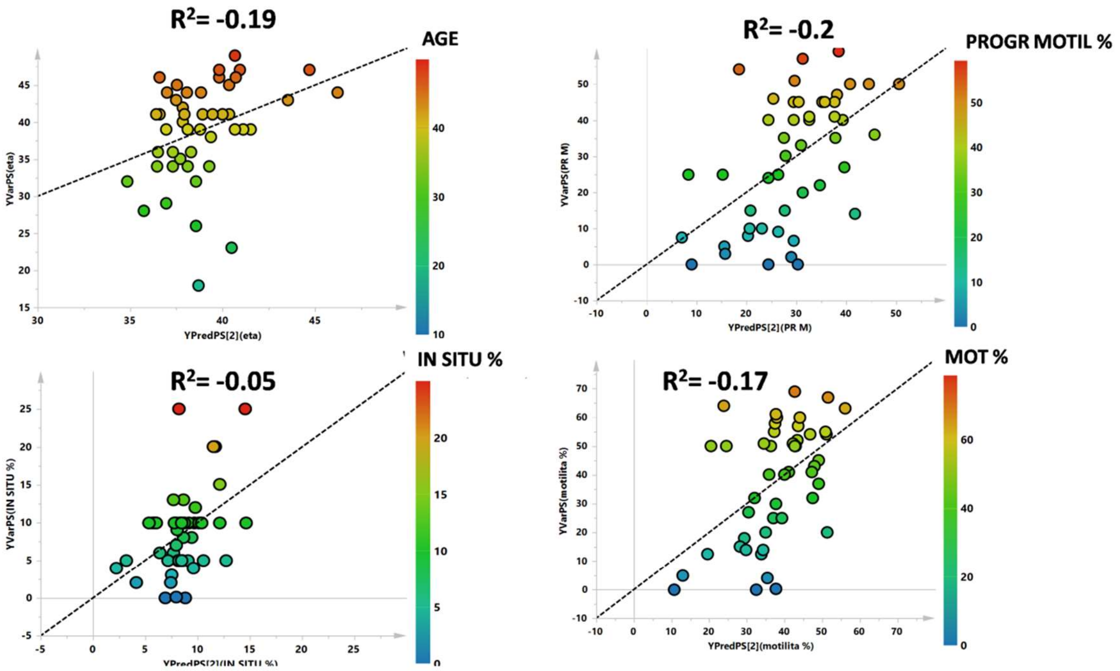

Figure S3. PLS correlation analysis between the metabolic profile of the patients and clinical parameters such as age, % of motility of spermatozoa , % progressive motility and % of “in situ” motility. Each point represent a seminal fluid sample of patients.
